# Disparities in deworming coverage between children with and without disabilities: insights from the DeWorm3 trial in India

**DOI:** 10.1136/bmjgh-2025-020531

**Published:** 2026-06-09

**Authors:** Shanquan Chen, Rohan Michael Ramesh, Kumudha Aruldas, Saravanakumar Puthupalayam Kaliappan, Bobeena Rachel Chandy, Beena Koshy, Smitha Jasper, Katherine E Halliday, Judd L Walson, William Oswald, Sitara Swarna Rao Ajjampur, Hannah Kuper

**Affiliations:** 1 School of Public Health, LKS Faculty of Medicine, The University of Hong Kong, Hong Kong, China; 2 International Centre for Evidence in Disability, London School of Hygiene and Tropical Medicine, London, UK; 3 The Wellcome Trust Research Laboratory, Division of Gastrointestinal Sciences and Center for Public Health, Christian Medical College Vellore, Vellore, India; 4 Department of Physical Medicine and Rehabilitation, Christian Medical College, Tamil Nadu, India; 5 Department of Developmental Paediatrics and Center for Public Health, Christian Medical College, Tamil Nadu, India; 6 Department of Ophthalmology, Christian Medical College, Tamil Nadu, India; 7 London School of Hygiene and Tropical Medicine Faculty of Infectious and Tropical Diseases, London, UK; 8 Departments of International Health, Medicine and Pediatrics, Johns Hopkins Bloomberg School of Public Health, Baltimore, Maryland, USA; 9 RTI International, Research Triangle Park, North Carolina, USA

**Keywords:** Global Health, Child health, Soil-transmitted helminth infections

## Abstract

**Background:**

Children with disabilities experience lower school enrolment rates and may be systematically excluded from school-based deworming programmes. The DeWorm3 trial provided an opportunity to evaluate whether community-wide mass drug administration (cMDA) could reduce these disparities by reaching children outside the education system.

**Methods:**

We conducted a secondary analysis of the data from the DeWorm3 trial in Tamil Nadu, India. Children aged 5–17 years (n=82 417) who participated in at least one of the six rounds of cMDA were included. Disability was assessed using the UNICEF/Washington Group’s Child Functioning Module. Mixed-effects logistic regression models examined associations between disability status and treatment coverage, adjusting for sociodemographic factors.

**Results:**

Children with disabilities (1.1% of the population) had higher unadjusted odds of not receiving deworming treatment compared with children without disabilities (OR=1.49, 95% CI 1.19 to 1.84), though this association was attenuated after adjustment (adjusted OR=1.10, 95% CI 0.86 to 1.37). Among school-attending children who received treatment, those with disabilities had significantly lower odds of receiving school-based treatment (adjusted OR=0.57, 95% CI 0.44 to 0.73). Subgroup analyses revealed that age, school attendance, parental marital status, geographical location and the COVID-19 pandemic significantly modified these associations.

**Conclusion:**

While cMDA reduced some disparities in deworming coverage, children with disabilities remained less likely to receive school-based treatment. Public health programmes should combine school-based approaches with targeted community outreach strategies to ensure equitable inclusion of children with disabilities, particularly those experiencing multiple vulnerabilities.

**WHAT IS ALREADY KNOWN ON THIS TOPIC**

**WHAT THIS STUDY ADDS**

- This study demonstrates that children with disabilities are significantly less likely to receive deworming treatment through schools than their typically developing peers, even when they are enrolled in school.
- The findings show that community-wide mass drug administration can reduce these disparities by reaching children outside the education system, though gaps persist for those with multiple vulnerabilities.

**HOW THIS STUDY MIGHT AFFECT RESEARCH, PRACTICE OR POLICY**

- Public health programmes should integrate school-based deworming with targeted community outreach and specialised distributor training to ensure the equitable inclusion of children with disabilities.
- Policy changes are needed to incorporate disability-inclusive metrics into the monitoring frameworks of mass drug administration campaigns to track and achieve global health equity goals.

## Introduction

 Globally, an estimated 200 million children aged 5–17 years live with disabilities.^1^ These children frequently face exclusions across multiple dimensions, including education and access to health interventions.^2 3^ Soil-transmitted helminths (STH) remain a major public health concern, particularly in low- and middle-income countries, where over 1.5 billion people are affected.^4^ Targeted deworming programmes are the cornerstone of STH control efforts aiming to reduce infection intensity and associated morbidity in risk groups through preventive chemotherapy.^4 5^ However, traditional school-based deworming (SBD) programmes, such as India’s National Deworming Day (NDD), primarily target preschool and school-aged children attending school, potentially excluding children with disabilities—many of whom are not enrolled in anganwadis (informal state-run learning centres for preschool children established under the Integrated Child Development Scheme) and school. Such systematic exclusions not only reinforce health inequities but may also hinder the overall success of elimination efforts.

The DeWorm3 trial represents a paradigm shift from STH control to evaluating the feasibility of STH transmission interruption through community-wide mass drug administration (cMDA) compared with SBD.^6 7^ cMDA aims to treat all individuals within a community, including children aged >1 year, regardless of school enrolment, thereby potentially addressing the limitations of school-based approaches. However, the effectiveness of cMDA in reaching vulnerable populations such as children with disabilities remains underexplored. This is an important topic as children with disabilities are disproportionately impacted by health disparities due to barriers in accessing care, heightened stigma and misconceptions regarding their health needs.^8^,^10^ The integration of disability-inclusive approaches within MDA campaigns is critical for achieving equitable health outcomes and sustaining transmission interruption goals.^4^ The paucity of evidence on deworming coverage among children with disabilities, particularly in community-based and school-based settings, underscores the need for targeted research.

This study aims to assess disparities in deworming treatment coverage between children with and without disabilities within the context of the DeWorm3 trial in southern India. Specifically, the study seeks to answer the following research questions: first, what is the likelihood of children with disabilities receiving deworming treatment compared with children without disabilities during cMDA? Second, among school-attending children who received deworming, what is the likelihood of children with disabilities being treated at school rather than in the community compared with children without disabilities? By addressing these questions, this study seeks to inform the design and implementation of equitable deworming strategies that include all children, particularly those with disabilities.

## Methods

### Study design and participants

This study is a secondary analysis of data from the DeWorm3 trial, a multicountry cluster-randomised community-based study designed to evaluate the feasibility of interrupting STH transmission through cMDA. Details of the trial design, methods and results have been described extensively elsewhere.^6 7^

The analysis focuses on intervention clusters in India, comprising 40 clusters in Tamil Nadu’s Timiri and Jawadhu Hills blocks.^511^,^14^ These clusters included a total population of approximately 141 000, residing in 36 536 households across 219 villages in Timiri and 154 villages in Jawadhu Hills.^511^,^14^ This data represents all 40 clusters in the study area. The residents in this geographical area are characterised by predominantly rural (77%) and tribal (23%) residents, with limited sanitation access (34.6%) and significant engagement in agricultural activities (20% in Timiri, 90% in Jawadhu Hills).^511^,^14^

Eligible participants for this analysis were children aged 5–17 years who had participated in at least one round of cMDA.

### Data collection

Data were collected as part of the routine activities of the DeWorm3 trial by adhering to rigorous and systematic protocols. The baseline census, conducted between 2017 and 2018, enumerated all households and individuals within the intervention clusters.^5 15^ This census captured extensive demographic, socio-economic and geographical data, including age, gender, disability status, education levels and household wealth indicators. Each household received a unique identification card with a barcode to enable precise tracking in subsequent data collection rounds. Annual updates to the census were undertaken to account for population changes such as births, deaths and migration, ensuring accurate denominators for coverage and treatment analyses.

During the six rounds of cMDA conducted for 3 years (2018–2020), albendazole was provided to eligible individuals aged 1–99 years. For children, deworming treatment was initially offered through the NDD programme administered by teachers at schools. Following NDD activities, community drug distributors (CDDs) conducted cMDA, which targeted individuals not treated during NDD (identifiable by the absence of ink marks on fingers). Trained CDDs, supported by study officers, delivered the drug directly to households. Treatment administration was directly observed and electronically recorded using a custom-designed treatment register. Efforts were made to maximise treatment coverage through repeated household visits for individuals initially unavailable. If absence persisted, treatment tablets were left with the head of the household, and acceptance was documented. For school-attending children, deworming during India’s NDD programme was verified by inspecting ink marks applied on their fingers during SBD sessions.

### Outcomes and measurement

The primary outcomes of this study were: (1) the disability-related disparity in treatment coverage during cMDA, measured as the likelihood of children with disabilities receiving treatment compared with children without disabilities, as recorded in cMDA treatment registers; and (2) the disparity in school-based treatment among dewormed, school-attending children, evaluated as the likelihood of children with disabilities receiving treatment at school compared with their typically developing peers. The latter outcome was assessed through self-reported data collected during surveys conducted after the first four cMDA rounds, as SBD was not implemented during the final two rounds due to the COVID-19 lockdown. During the second COVID-19 round, NDD activities were conducted in community settings rather than schools.

Disability status was assessed using the Child Functioning Module (CFM), a tool validated for use in surveys with mothers or primary caregivers as proxy respondents.^16^ Developed by UNICEF in collaboration with the Washington Group on Disability Statistics, CFM is grounded in the WHO’s International Classification of Functioning and the biopsychosocial model of disability.^17^ The module has undergone extensive expert review and testing across multiple countries to ensure the quality of its questions and their cultural appropriateness for respondents with varying linguistic backgrounds and experiences of disability.^17^ For children aged 5–17 years, the CFM evaluates functional difficulties across several domains, including vision/seeing, hearing, mobility/walking, self-care, communication/comprehension, learning, remembering, focusing attention/concentrating, coping with change, controlling behaviour and relationships/making friends. Details of the module’s structure and administration are described elsewhere.^18^ Functional difficulties in each domain were measured using a four-point response scale: ‘no difficulty’, ‘some difficulty’, ‘a lot of difficulty’ or ‘cannot do at all’. Following UNICEF’s definition, children were classified as having a functional disability if they were reported to have ‘a lot of difficulty’ or ‘cannot do at all’ in at least one domain.^16^

### Covariates

The following variables were considered as covariates: individual-level sociodemographic factors, such as age, gender and school attendance, as well as household-level characteristics, including the highest education level attained within the family, parental marital status and household size (≥5 members). Additional factors considered were caste, rural residence (yes/no), study site, distance to schools and survey round (1–6), to account for geographical and temporal variations in the analysis.

### Statistical analysis

Categorical variables were reported as frequencies and percentages, while continuous variables were reported as means with SD. Differences in outcomes were evaluated using two-tailed t-tests for continuous variables and χ² tests for categorical variables.

A mixed-effects logistic regression model was employed to investigate the association between disability status and likelihood of not receiving deworming treatment. The dependent variable was untreated status (yes or no), with disability status (yes or no) as the primary independent variable. The model was adjusted for sociodemographic covariates, including age, sex, school attendance, household educational attainment, parental marital status, household size, socio-economic status, caste, residential location, study site and survey round. Random intercepts were included for participants’ unique IDs to account for within-individual clustering. Results are reported as ORs with 95% CIs. Subgroup analyses were conducted by repeating the regression model stratified by covariates, and the interaction effects between disability status and subgroup variables were assessed using the interaction terms within the overall model.

The analysis was repeated to examine the association between disability status and the likelihood of receiving SBD treatment among children who attended school and had received deworming.

Missing data were predominantly observed in variables such as age (0.23%), gender (0.01%), school attendance (0.24%), religion (1.81%), caste (2.20%), socio-economic status (<0.01%), household education (0.03%), parental marital status (<0.01%) and household size (0.23%). To minimise bias due to missing data, multiple imputations using chained equations with imputations were performed.

All statistical analyses were conducted using R software (V.4.3.0), with statistical significance set at p<0.05.

The Euclidean distance to schools the children attended was analysed using ArcGIS Pro (V. 3.3.2)

### Patient and public involvement

This study is a secondary analysis of data collected during the DeWorm3 trial. While patients and the public were not involved in the design, analysis or dissemination of this specific secondary analysis, the broader DeWorm3 trial incorporated community engagement as a core component. Community leaders, parents and local health stakeholders were actively engaged during trial planning and implementation to ensure culturally appropriate delivery and acceptability of MDA activities. CDDs, drawn from the local population, played a central role in treatment delivery and community sensitisation. The study team also conducted formative qualitative research to understand local perspectives and incorporated these findings into the implementation strategy. Although children with disabilities and their caregivers were not specifically involved in shaping this analysis, our findings highlight the importance of future studies incorporating participatory approaches to address equity in neglected tropical disease programmes.

## Results

Table 1 presents the characteristics of 82 417 children aged 5–17 years from the DeWorm3 trial conducted in India. Among these children, 58.8% were aged 12–17 years, 47.8% were female and 89.9% had attended school. Most children (93.2%) received deworming treatment, with about half being treated in school and remaining half in the community. Children with disabilities constituted 1.1% of the total population, with varying types of functional difficulties, including seeing (0.1%), hearing (0.1%), mobility/walking (0.4%), self-care (0.4%), communication (0.5%) and others. The majority of children came from households where parents were married (90.3%), lived in rural areas (85.2%) and belonged to the Hindu religion (96.3%). The study population was primarily from the Timiri site (71.9%) compared with Jawadhu Hills (28.1%). During the COVID-19 pandemic period (rounds 5–6), there was a notable shift from school-based to community-based treatment delivery, with virtually no school-based treatments during these rounds. The basic description by outcomes was also presented in table 1.

**Table 1:** Basic characteristics, by deworm treatment status and by treated place

	All (n=82 417)	Not treated (n=5625)	Treated (n=76 792)	P value	Treated in school (n=38 430)	Treated in community (n=38 362)	P value
Age							
5–11 years	33 959 (41.2)	2070 (36.8)	31 889 (41.5)	<0.01	16 411 (42.7)	15 478 (40.3)	<0.01
12–17 years	48 458 (58.8)	3555 (63.2)	44 903 (58.5)		22 019 (57.3)	22 884 (59.7)	
Sex (= female)	39 411 (47.8)	2578 (45.8)	36 833 (48.0)	<0.01	19 198 (50.0)	17 635 (46.0)	<0.01
Attending school (= yes)	74 123 (89.9)	4165 (74.0)	69 958 (91.1)	<0.01	37 153 (96.7)	32 805 (85.5)	<0.01
Highest education degree of family members							
No education	19 515 (23.7)	2340 (41.6)	17 175 (22.4)	<0.01	8057 (21.0)	9118 (23.8)	<0.01
Any primary	15 241 (18.5)	904 (16.1)	14 337 (18.7)		7252 (18.9)	7085 (18.5)	
Any middle	17 589 (21.3)	881 (15.7)	16 708 (21.8)		8711 (22.7)	7997 (20.8)	
Any secondary or higher	30 072 (36.5)	1500 (26.7)	28 572 (37.2)		14 410 (37.5)	14 162 (36.9)	
Parents marital status							
Married	74 447 (90.3)	4865 (86.5)	69 582 (90.6)	<0.01	34 971 (91.0)	34 611 (90.2)	<0.01
Previously or never married	7970 (9.7)	760 (13.5)	7210 (9.4)		3459 (9.0)	3751 (9.8)	
Household size (≥5)	49 703 (60.3)	3550 (63.1)	46 153 (60.1)	<0.01	23 396 (60.9)	22 757 (59.3)	<0.01
Member in the family temporarily migrated (=yes)	504 (0.6)	496 (8.8)	8 (0.0)	<0.01	6 (0.0)	2 (0.0)	0.29
Distance to school (≤1 km)		–	34 792 (49.7)		18 544 (49.9)	16 248 (49.5)	0.31
Household socio-economic status							
Poor (Lower three quintiles)	49 993 (60.7)	2295 (40.8)	47 698 (62.1)	<0.01	24 057 (62.6)	23 641 (61.6)	0.01
Rich (Upper two quintiles)	32 424 (39.3)	3330 (59.2)	29 094 (37.9)		14 373 (37.4)	14 721 (38.4)	
Religion (= Hinduism (India))	79 367 (96.3)	5407 (96.1)	73 960 (96.3)	0.49	37 142 (96.6)	36 818 (96.0)	<0.01
Caste							
Most backward caste	19 279 (23.4)	884 (15.7)	18 395 (24.0)	<0.01	9596 (25.0)	8799 (22.9)	<0.01
Scheduled tribes	22 362 (27.1)	2848 (50.6)	19 514 (25.4)		9372 (24.4)	10 142 (26.4)	
Scheduled caste	20 510 (24.9)	909 (16.2)	19 601 (25.5)		9863 (25.7)	9738 (25.4)	
Backward caste	19 534 (23.7)	950 (16.9)	18 584 (24.2)		9266 (24.1)	9318 (24.3)	
Higher caste	732 (0.9)	34 (0.6)	698 (0.9)		333 (0.9)	365 (1.0)	
Residence place (= rural)	70 251 (85.2)	4891 (87.0)	65 360 (85.1)	<0.01	32 938 (85.7)	32 422 (84.5)	<0.01
Site							
Timiri	59 286 (71.9)	2739 (48.7)	56 547 (73.6)	<0.01	28 692 (74.7)	27 855 (72.6)	<0.01
Jawadhu Hills	23 131 (28.1)	2886 (51.3)	20 245 (26.4)		9738 (25.3)	10 507 (27.4)	
Disabled (= Yes)	940 (1.1)	92 (1.6)	848 (1.1)	<0.01	264 (0.7)	584 (1.5)	<0.01
Seeing	92 (0.1)	11 (0.2)	81 (0.1)	0.08	30 (0.1)	51 (0.1)	0.03
Hearing	106 (0.1)	20 (0.4)	86 (0.1)	<0.01	24 (0.1)	62 (0.2)	<0.01
Mobility/walking	327 (0.4)	44 (0.8)	283 (0.4)	<0.01	58 (0.2)	225 (0.6)	<0.01
Self-care	329 (0.4)	43 (0.8)	286 (0.4)	<0.01	45 (0.1)	241 (0.6)	<0.01
Communication	381 (0.5)	54 (1.0)	327 (0.4)	<0.01	69 (0.2)	258 (0.7)	<0.01
Learning	321 (0.4)	43 (0.8)	278 (0.4)	<0.01	56 (0.1)	222 (0.6)	<0.01
Remembering	304 (0.4)	48 (0.9)	256 (0.3)	<0.01	44 (0.1)	212 (0.6)	<0.01
Concentrating	268 (0.3)	45 (0.8)	223 (0.3)	<0.01	31 (0.1)	192 (0.5)	<0.01
Coping with change	248 (0.3)	39 (0.7)	209 (0.3)	<0.01	29 (0.1)	180 (0.5)	<0.01
Behaviour controlling	295 (0.4)	42 (0.7)	253 (0.3)	<0.01	47 (0.1)	206 (0.5)	<0.01
Making friends	268 (0.3)	43 (0.8)	225 (0.3)	<0.01	36 (0.1)	189 (0.5)	<0.01
Survey round							
Round 1	11 562 (14.0)	851 (15.1)	10 711 (13.9)	<0.01	7499 (19.5)	3212 (8.4)	<0.01
Round 2	12 728 (15.4)	555 (9.9)	12 173 (15.9)	<0.01	9683 (25.2)	2490 (6.5)	<0.01
Round 3	13 831 (16.8)	441 (7.8)	13 390 (17.4)	<0.01	10 481 (27.3)	2909 (7.6)	<0.01
Round 4	14 406 (17.5)	382 (6.8)	14 024 (18.3)	<0.01	10 725 (27.9)	3299 (8.6)	<0.01
Round 5	14 937 (18.1)	2240 (39.8)	12 697 (16.5)	<0.01	41 (0.1)	12 656 (33.0)	<0.01
Round 6	14 953 (18.1)	1156 (20.6)	13 797 (18.0)	<0.01	1 (0.0)	13 796 (36.0)	<0.01
COVID-19 pandemic (= yes)	29 890 (36.3)	3396 (60.4)	26 494 (34.5)	<0.01	42 (0.1)	26 452 (69.0)	<0.01

Data was reported as the mean (SD) or number (percentage).

P values were derived using two-tailed tests for continuous variables and two-tailed χ² tests for categorical variables.

For categorical variables with expected cell counts less than 5, Fisher’s exact test was employed.

‘-’ no corresponding data.

**Figure 1:**
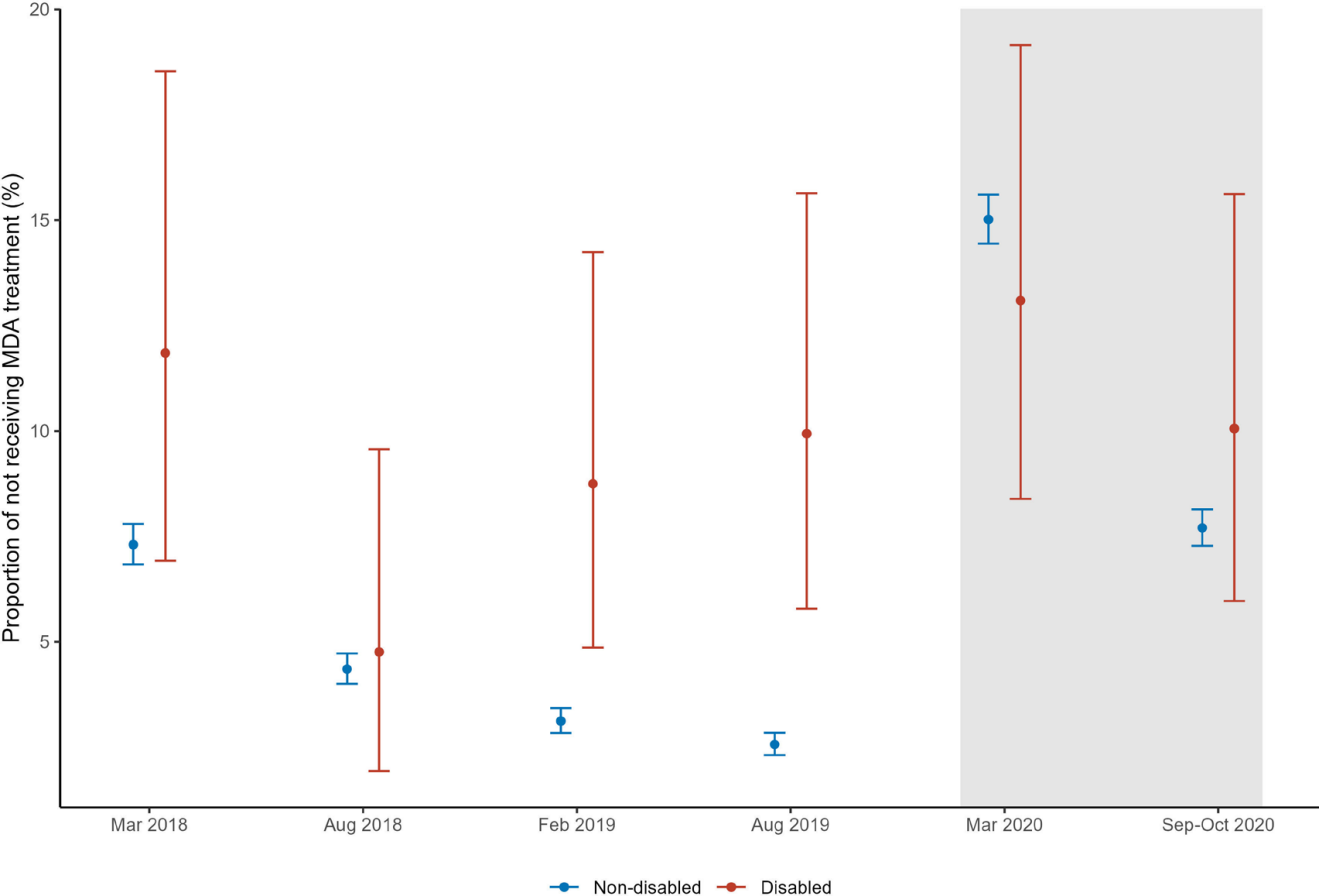
Proportion of not receiving MD treatment, by survey round and disability status. Points show the percentage, while vertical lines show the corresponding 95% CIs. The shaded grey area denotes the period during which data were collected amid the COVID-19 pandemic. MD, mass drug, MDA, mass drug administration.

**Figure 2:**
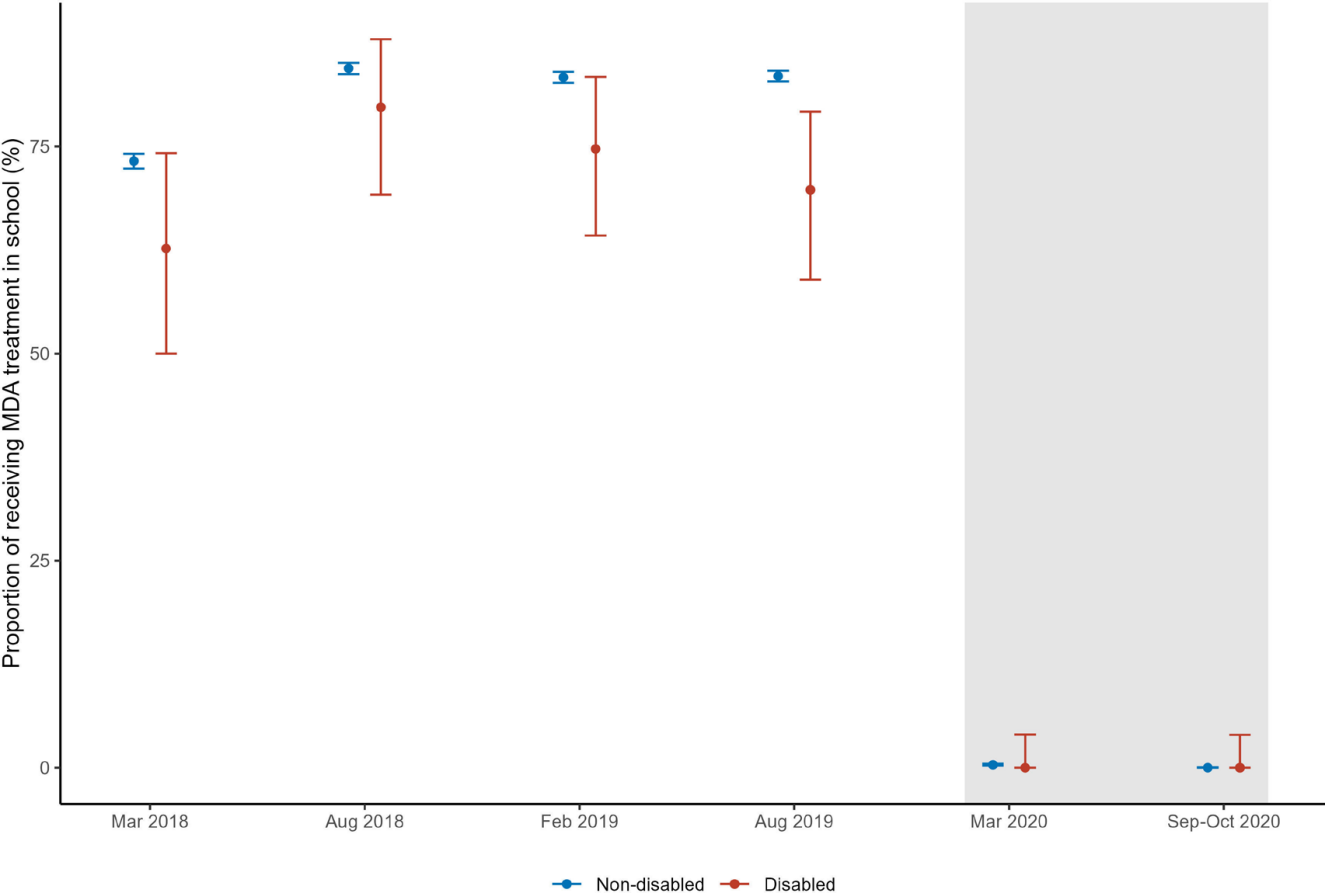
Proportion of receiving MD treatment in school, by survey round and disability status. Points show the percentage, while vertical lines show the corresponding 95% CIs. The shaded grey area denotes the period during which data were collected amid the COVID-19 pandemic. MD, mass drug, MDA, mass drug administration.

Figure 1 and figure 2 show the treatment coverage patterns across the survey rounds for children with and without disabilities. Across all survey rounds, the proportion of children without disabilities who did not receive MDA treatment ranged from approximately 2.5% to 15.0%, whereas the corresponding proportion of children with disabilities ranged from approximately 5.0% to 13.0%. Prior to the COVID-19 pandemic (2018–2019), untreated proportions were consistently low among children without disabilities (generally below 8%), while children with disabilities exhibited persistently higher levels of non-treatment, often approaching or exceeding 10% (figure 1). During the COVID-19 survey rounds in 2020, the proportion of untreated children increased in both groups, with a more pronounced rise among children without disabilities, reaching approximately 15% in March 2020 (figure 1). Figure 2 shows that among school-attending children who received treatment, those with disabilities were less likely to receive school-based treatment (60–75% vs 75–85%) during rounds 1–4 and instead were treated in the community, with treatment at schools ceasing for both groups during COVID-19 rounds 5–6.

Table 2 presents the results of mixed-effects logistic regression analysis examining associations between disability status and deworming treatment non-receipt. While unadjusted analysis indicated children with disabilities had significantly higher odds of being untreated (OR=1.49, 95% CI 1.19 to 1.84), this association was attenuated and became non-significant after adjusting for sociodemographic covariates including age, sex, school attendance, household educational attainment, parental marital status, household size, socio-economic status, caste, residential location, site and survey round (adjusted OR=1.10, 95% CI 0.86 to 1.37). Subgroup analyses revealed significant interaction effects across several variables. Age demonstrated a significant interaction (interaction adjusted OR=2.05, 95% CI 1.20 to 3.66), with older children (12–17 years) with disabilities showing disproportionately higher odds of non-treatment. School attendance also exhibited a significant interaction effect (interaction adjusted OR=1.66, 95% CI 1.02 to 2.80), indicating that among school attendees, disability status was associated with increased odds of non-treatment. Parental marital status was another significant modifier (interaction adjusted OR=1.80, 95% CI 1.00 to 3.15), with children with disabilities from previously or never married parents showing elevated odds of non-treatment. Geographical variation was evident through a significant site interaction (interaction adjusted OR=0.50, 95% CI 0.31 to 0.81), suggesting differential treatment patterns between regions. The pandemic period demonstrated a significant interaction effect (interaction adjusted OR=0.39, 95% CI 0.25 to 0.62), specifically showing that during the COVID-19 pandemic, the likelihood of non-treatment for children with disabilities was significantly reduced.

**Table 2:** Association between disability and non-receipt of deworming treatment, by overall and subgroups

Outcomes	Children with disability n/N (%)	Children without disability n/N (%)	Unadjusted OR	Adjusted OR	Interaction with subgroup	
					Unadjusted OR	Adjusted OR
Overall	92/940 (9.8)	5533/81 477 (6.8)	1.49 (1.19 to 1.84)***	1.10 (0.86 to 1.37)	–	–
By age						
5–11 years	20/329 (6.1)	2050/33 630 (6.1)	1.00 (0.61 to 1.53)	0.72 (0.43 to 1.15)	Ref	Ref
12–17 years	72/611 (11.8)	3483/47 847 (7.3)	1.70 (1.32 to 2.17)***	1.30 (0.99 to 1.68)*	1.71 (1.04 to 2.93)*	2.05 (1.20 to 3.66)*
By gender						
Females	45/443 (10.2)	2533/38 968 (6.5)	1.63 (1.18 to 2.19)**	1.14 (0.81 to 1.58)	Ref	Ref
Males	47/497 (9.5)	3000/42 509 (7.1)	1.38 (1.00 to 1.84)*	1.02 (0.73 to 1.40)	0.85 (0.55 to 1.31)	0.86 (0.54 to 1.36)
Attended school						
Yes	25/525 (4.8)	4140/73 598 (5.6)	0.84 (0.55 to 1.23)	0.79 (0.50 to 1.17)	Ref	Ref
No	67/415 (16.1)	1393/7879 (17.7)	0.90 (0.68 to 1.16)	1.22 (0.90 to 1.61)	1.07 (0.67 to 1.76)	1.66 (1.02 to 2.80)*
Highest education degree of family members						
No education	36/295 (12.2)	2304/19 220 (12)	1.02 (0.71 to 1.43)	0.99 (0.67 to 1.43)	Ref	Ref
Any primary	24/212 (11.3)	880/15 029 (5.9)	2.05 (1.30 to 3.09)***	1.35 (0.84 to 2.10)	2.01 (1.14 to 3.49)*	1.45 (0.80 to 2.59)
Any middle	8/150 (5.3)	873/17 439 (5)	1.07 (0.48 to 2.05)	0.83 (0.36 to 1.63)	1.05 (0.44 to 2.22)	0.90 (0.37 to 1.95)
Any secondary or higher	24/283 (8.5)	1476/29 789 (5)	1.78 (1.14 to 2.65)**	1.04 (0.64 to 1.60)	1.74 (1.00 to 3.00)*	1.18 (0.65 to 2.09)
Parents marital status						
Married	72/815 (8.8)	4793/73 632 (6.5)	1.39 (1.08 to 1.76)**	0.98 (0.75 to 1.26)	Ref	Ref
Previously or never married	20/125 (16)	740/7845 (9.4)	1.83 (1.10 to 2.90)*	1.65 (0.96 to 2.72).	1.31 (0.75 to 2.22)	1.80 (1.00 to 3.15)*
Household socio-economic status						
Rich (Upper two quintiles)	45/413 (10.9)	3285/32 011 (10.3)	1.07 (0.77 to 1.44)	0.91 (0.64 to 1.25)	Ref	Ref
Poor (Lower three quintiles)	47/527 (8.9)	2248/49 466 (4.5)	2.06 (1.50 to 2.75)***	1.25 (0.89 to 1.71)	0.52 (0.34 to 0.80)**	0.65 (0.41 to 1.03)
Residence place						
Urban	10/100 (10)	724/12 066 (6)	1.74 (0.85 to 3.20).	1.36 (0.64 to 2.59)	Ref	Ref
Rural	82/840 (9.8)	4809/69 411 (6.9)	1.45 (1.15 to 1.82)***	1.08 (0.84 to 1.37)	1.20 (0.56 to 2.30)	1.08 (0.49 to 2.14)
Site						
Timiri	59/624 (9.5)	2680/58 662 (4.6)	2.18 (1.65 to 2.83)***	1.22 (0.91 to 1.61)	Ref	Ref
Jawadhu Hills	33/316 (10.4)	2853/22 815 (12.5)	0.82 (0.56 to 1.15)	0.73 (0.48 to 1.06)	0.37 (0.24 to 0.58)***	0.50 (0.31 to 0.81)**
Pandemic						
Year ≤2019	53/603 (8.8)	2176/51 924 (4.2)	2.20 (1.64 to 2.90)***	1.64 (1.20 to 2.18)***	Ref	Ref
Year ≥2020	39/337 (11.6)	3357/29 553 (11.4)	1.02 (0.72 to 1.41)	0.70 (0.48 to 0.99)*	0.46 (0.30 to 0.72)***	0.39 (0.25 to 0.62)***

A mixed-effects logistic regression analysis examining the association between disability status and non-receipt of deworming treatment.

The model fitted with non-receipt of deworming treatment status (yes or no) as the dependent variable and disability status (yes or no (reference)) as the primary predictor, with adjustments for sociodemographic covariates including age, sex, school attendance, household educational attainment, parental marital status, household size, socio-economic status, caste, residential location, site and survey round. Random intercepts were included for participant’s unique ID.

Results are presented as ORs with corresponding 95% CIs.

Analyses include overall population estimates and stratified subgroup analyses. Interaction effects between disability status (non-disabled children as reference) and subgroup variables were assessed by including the interactive term between disability status and corresponding subgroup variable.

n/N refers to the number of untreated children (n) out of the total number of children in that category (N). For example, 92/940 indicates that 92 children with disability out of a total of 940 children with disability did not receive deworming treatment.

p>0.5, *p<0.05, **p<0.01, ***p<0.001.

Table 3 presents the results of mixed-effects logistic regression analysis examining the associations between disability status and deworming treatment location among children who attended school and had received treatment. Overall, children with disabilities had significantly lower odds of receiving school-based treatment compared with children without disabilities (adjusted OR=0.57, 95% CI 0.44 to 0.73) after adjusting for covariates. Subgroup analyses identified several significant interaction effects. Age emerged as a strong effect modifier (interaction adjusted OR=3.31, 95% CI 1.99 to 5.59), indicating that older children (12–17 years) with disabilities had substantially higher odds of receiving school-based treatment compared with younger children with disabilities. Parental marital status demonstrated a significant interaction effect (interaction adjusted OR=0.36, 95% CI 0.18 to 0.73), suggesting that children with disabilities whose parents were previously or never married had markedly lower odds of receiving school-based treatment compared with those with married parents. Distance to school also showed a significant interaction effect (interaction adjusted OR=0.45, 95% CI 0.26 to 0.77), with children with disabilities living within 1 km of school having lower odds of receiving school-based treatment compared with those living farther away.

**Table 3:** Association between disability status and treated in school, by overall and subgroups

Outcomes	Children with disability n/N (%)	Children without disability n/N (%)	Unadjusted OR	Adjusted OR	Interaction with subgroup	
					Unadjusted OR	Adjusted OR
Overall	230/319 (72.1)	36 881/45 245 (81.5)	0.59 (0.46 to 0.75)***	0.57 (0.44 to 0.73)***	–	–
By age						
5–11 years	82/137 (59.9)	16 214/19 580 (82.8)	0.31 (0.22 to 0.44)***	0.31 (0.22 to 0.45)***	Ref	Ref
12–17 years	148/182 (81.3)	20 667/25 665 (80.5)	1.05 (0.73 to 1.55)	0.96 (0.67 to 1.43)	3.40 (2.06 to 5.69)***	3.31 (1.99 to 5.59)***
By gender						
Females	121/163 (74.2)	18 474/21 969 (84.1)	0.55 (0.39 to 0.78)***	0.53 (0.37 to 0.76)***	Ref	Ref
Males	109/156 (69.9)	18 407/23 276 (79.1)	0.61 (0.44 to 0.87)**	0.60 (0.43 to 0.86)**	1.13 (0.69 to 1.84)	1.13 (0.68 to 1.86)
Highest education degree of family members						
No education	71/103 (68.9)	7482/9116 (82.1)	0.48 (0.32 to 0.75)***	0.50 (0.33 to 0.79)**	Ref	Ref
Any primary	50/63 (79.4)	6920/8378 (82.6)	0.81 (0.45 to 1.56)	0.80 (0.44 to 1.55)	1.67 (0.81 to 3.61)	1.59 (0.76 to 3.46)
Any middle	41/55 (74.5)	8407/10 062 (83.6)	0.58 (0.32 to 1.10)	0.55 (0.30 to 1.06)	1.19 (0.57 to 2.55)	1.06 (0.50 to 2.29)
Any secondary or higher	68/98 (69.4)	14 072/17 689 (79.6)	0.58 (0.38 to 0.91)*	0.55 (0.36 to 0.87)**	1.20 (0.66 to 2.20)	1.11 (0.60 to 2.05)
Parents marital status						
Married	210/280 (75)	33 612/41 246 (81.5)	0.68 (0.52 to 0.90)**	0.66 (0.50 to 0.87)**	Ref	Ref
Previously or never married	20/39 (51.3)	3269/3999 (81.7)	0.24 (0.12 to 0.45)***	0.24 (0.12 to 0.47)***	0.34 (0.17 to 0.69)**	0.36 (0.18 to 0.73)**
Household socio-economic status						
Rich (Upper two quintiles)	138/185 (74.6)	23 351/28 952 (80.7)	0.70 (0.51 to 0.99)*	0.65 (0.47 to 0.92)*	Ref	Ref
Poor (Lower three quintiles)	92/134 (68.7)	13 530/16 293 (83)	0.45 (0.31 to 0.65)***	0.48 (0.33 to 0.70)***	0.64 (0.39 to 1.04)	0.70 (0.42 to 1.15)
Distance to school						
≤1 km	132/194 (68)	18 422/22 199 (83)	0.44 (0.32 to 0.60)***	0.42 (0.31 to 0.57)***	0.48(0.28, 0.81)**	0.45(0.26, 0.77)**
>1 km	98/125 (78.4)	18 459/23 046 (80.1)	0.90 (0.60 to 1.41)	0.89 (0.58 to 1.39)	Ref	Ref
Residence place						
Rural	209/281 (74.4)	31 495/38 323 (82.2)	0.63 (0.48 to 0.83)***	0.62 (0.48 to 0.82)***	Ref	Ref
Urban	21/38 (55.3)	5386/6922 (77.8)	0.35 (0.19 to 0.68)***	0.32 (0.17 to 0.63)***	0.56 (0.28 to 1.13)	0.53 (0.26 to 1.08).
Site						
Timiri	153/207 (73.9)	27 809/34 129 (81.5)	0.64 (0.47 to 0.89)**	0.61 (0.45 to 0.84)**	Ref	Ref
Jawadhu Hills	77/112 (68.8)	9072/11 116 (81.6)	0.50 (0.33 to 0.75)***	0.53 (0.35 to 0.81)**	0.77 (0.46 to 1.29)	0.77 (0.46 to 1.30)

A mixed-effects logistic regression analysis examining the association between disability status and deworming treated in school or community.

The model fitted with treated in school (yes or no) as the dependent variable and disability status (yes or no (reference)) as the primary predictor, with adjustments for sociodemographic covariates including age, sex, household educational attainment, parental marital status, household size, socio-economic status, caste, residential location, site and survey round. Random intercepts were included for participant’s unique ID.

Results are presented as ORs with corresponding 95% CIs.

Analyses include overall population estimates and stratified subgroup analyses. Interaction effects between disability status (non-disabled children as reference) and subgroup variables were assessed by including the interactive term between disability status and corresponding subgroup variable.

n/N refers to the number of children treated in school (n) out of the total number of children who received deworming treatment in that category (N). For example, 230/319 indicates that 230 children with disability out of a total of 319 treated children with disability received their treatment in school.

p>0.5, *p<0.05, **p<0.01, ***p<0.001.

## Discussion

### Principle findings

This study highlights significant disparities in deworming treatment coverage between children with and without disabilities in the context of cMDA and SBD. Overall, children with disabilities were less likely to receive deworming treatment compared with their typically developing peers, particularly during SBD campaigns. While cMDA demonstrated relatively higher inclusivity by reaching children with disabilities outside the school system, treatment gaps persisted, underscoring systemic barriers to equitable healthcare access. These disparities were exacerbated by contextual factors such as age, parental marital status, distance to school and geographical region, with older children, those from previously or never-married parents and those residing in certain rural clusters experiencing the greatest inequities and the least likelihood of being dewormed. Additionally, during the COVID-19 pandemic, treatment patterns were substantially modified, with the impact of treatment access disruption being more pronounced among typically developing children compared with children with disabilities, revealing complex shifts in healthcare delivery during the pandemic period. These findings emphasise the need for targeted strategies to ensure equitable inclusion of children with disabilities in deworming campaigns, both in schools and the broader community.

### Compare with previous studies

Our findings support the concern that school-based health programmes systematically exclude children with disabilities, thereby perpetuating health inequalities and potentially compromising the effectiveness of public health campaigns. School-based delivery platforms constitute essential mechanisms for providing preventive health interventions for children, including deworming, nutritional supplementation, immunisation and vision screening.^19^,^21^ However, these approaches inherently disadvantage children with disabilities, who experience significantly lower rates of school enrolment and attendance compared with their non-disabled peers.^22^ This systematic exclusion contributes to measurable health disparities, as demonstrated in studies where school-based health services represented primary access points for preventive care.^23 24^ For instance, research has documented how children with disabilities miss crucial nutritional interventions delivered through schools, subsequently exhibiting higher rates of malnutrition and associated health complications.^25^ Multiple investigations across varied contexts have confirmed that children with disabilities face disproportionate barriers to healthcare access,^26 27^ with school-based exclusion representing a significant contributing factor. The persistent marginalisation of children with disabilities from school-based health interventions not only exacerbates existing health inequities, but may also undermine population-level public health initiatives by preventing the achievement of coverage thresholds necessary for intervention success.

Nevertheless, we found that gaps in treatment access for children with disabilities can be effectively addressed through community-based outreach efforts. Our findings demonstrate that cMDA approaches achieved higher inclusivity for children with disabilities compared with SBD programmes, challenging persistent assumptions that children with disabilities are inherently unreachable or hidden within communities. This observation aligns with emerging evidence suggesting that targeted community interventions can successfully mitigate the healthcare disparities experienced by marginalised populations.^28^ While the primary objective remains expanding educational inclusion for children with disabilities—thereby facilitating access to both academic opportunities and associated health interventions—the reality necessitates complementary approaches. Integrating community outreach with school-based programmes creates a comprehensive framework that ensures that health services reach children with disabilities, regardless of educational enrolment status. This dual-channel strategy not only promotes equitable health outcomes but also strengthens overall intervention effectiveness by capturing populations that would otherwise remain excluded. The successful implementation of community-wide deworming in our study demonstrates the feasibility of inclusive public health approaches that accommodate the needs of children with disabilities without requiring substantial infrastructure modifications or resource allocation.

The intersectional nature of disability and various social determinants substantially influences healthcare access patterns among children with disabilities. These children represent a remarkably diverse population across dimensions of age, gender, socio-economic status and geographical location, all of which are established determinants of health outcomes.^29^,^31^ The significant site interaction effect (interaction adjusted OR=0.50, 95% CI 0.31 to 0.81) highlights how geographic context shapes disability-related treatment disparities. In Timiri, children with disabilities had notably higher odds of non-treatment compared to their non-disabled peers, whereas in Jawadhu Hills this disparity was absent, and disabled children were, if anything, slightly better reached. This pattern may reflect differences in community drug distributor practices, community cohesion, or population density between the two sites, and warrants further investigation. While the importance of examining intersectional factors in healthcare access is increasingly recognised, many studies remain underpowered to detect these complex interactions, resulting in limited evidence. Our research contributes significantly to this knowledge gap through robust assessment of effect modification across multiple dimensions. The findings reveal pronounced roles for social factors in mediating treatment access, including parental marital status and distance to school, alongside child-specific characteristics such as age. This pattern underscores the critical function of caregivers in facilitating healthcare access for children with disabilities, suggesting that effective interventions must extend beyond child-focused approaches to include caregiver support and education regarding preventive healthcare importance. The heterogeneity in treatment coverage observed across different subgroups challenges simplistic approaches to disability inclusion and advocates for nuanced strategies that address the multifaceted barriers experienced by diverse segments within this population.^32^,^35^

The implications of our findings extend to both local and global contexts, necessitating targeted policy adjustments and programme innovations. For local policymakers and programme implementers in India, this research underscores the importance of redesigning the NDD initiative to incorporate robust community outreach components that specifically address children with disabilities who remain outside the educational system. Health workers and CDDs should receive specialised training on disability inclusion, with particular emphasis on identifying and reaching households with children with disabilities. Furthermore, coordination between education and health ministries must be strengthened to develop comprehensive databases that capture children with disabilities, regardless of school enrolment status. Globally, these findings hold significant relevance for other MDA programmes targeting various neglected tropical diseases in low- and middle-income countries where similar exclusion patterns are likely to exist. International organisations and funding bodies supporting such initiatives should incorporate disability-inclusive metrics into their monitoring frameworks and establish conditional funding mechanisms that incentivise inclusive programming. Additionally, the demonstrated effectiveness of community-wide approaches offers a valuable model for addressing health inequities beyond deworming—including vaccination campaigns, nutritional interventions and other preventive services—potentially transforming how public health initiatives conceptualise and operationalise inclusivity for marginalised populations.

### Strength and limitations

This study has several strengths. It is one of the first studies to comprehensively evaluate disparities in deworming coverage among children with and without disabilities, leveraging high-quality, systematically collected data from the DeWorm3 trial, a large-scale, multicountry randomised study. The use of the CFM, a validated tool grounded in the biopsychosocial model of disability, provides robust and standardised assessments of functional difficulty across multiple domains, ensuring the reliability of disability classification. Additionally, the inclusion of both cMDA and SBD programmes allows for an advanced comparison of these delivery platforms, shedding light on equity gaps in access to health interventions.

However, this study has limitations. First, the reliance on caregiver-reported data on disability through the CFM may have introduced reporting bias, particularly in communities where stigma around disability is prevalent. Moreover, the CFM uses a relatively high threshold of ‘a lot’ of difficulty or more in at least one domain, and so may not capture all the children with disabilities. Second, while the study adjusted for a range of covariates, residual confounding may persist, especially for unmeasured factors, such as household attitudes toward disability or community-level barriers. Third, the generalisability of findings may be limited to settings similar to the DeWorm3 trial sites, particularly rural and tribal populations in Tamil Nadu, India. Finally, the impact of the COVID-19 pandemic on treatment coverage, while an important finding, complicates direct comparisons between school-based and community-based approaches, as the latter became the primary mode of treatment delivery during pandemic-related disruptions.

## Conclusion

School-based health programmes may exclude children with disabilities, as evidenced by disparities in deworming coverage in southern India. While cMDA showed promise in reducing these inequities, gaps persisted, particularly for children with disabilities from specific demographic and geographical subgroups. The findings underscore the importance of combining school-based approaches with targeted community outreach to ensure comprehensive coverage. To achieve equitable health outcomes and successful STH transmission interruption, deworming programmes must adopt disability-inclusive strategies. Future public health interventions should incorporate disability metrics in monitoring frameworks and design delivery approaches that accommodate children with diverse functional needs and living contexts.

## Supplementary material

10.1136/bmjgh-2025-020531 online supplemental file 1

## Data Availability

Data are available upon reasonable request.
