# Supplementary material for: Disparities in deworming coverage between children with and without disabilities: insights from the DeWorm3 trial in India
Source: BMJ Glob Health. 2026 Jun 9;11(6):e020531. doi: 10.1136/bmjgh-2025-020531 (PMC13264863; doi:10.1136/bmjgh-2025-020531)
Supplement: online supplemental file 1 [file bmjgh-11-6-s001.docx]

### BMJ Global Health Author Reflexivity Statement

Adapted from Morton, B., Vercueil, A., Masekela, R., Heinz, E., Reimer, L., Saleh, S., Kalinga, C., Seekles, M., Biccard, B., Chakaya, J., Abimbola, S., Obasi, A. and Oriyo, N. (2022), Consensus statement on measures to promote equitable authorship in the publication of research from international partnerships. Anaesthesia, 77: 264-276. <https://doi.org/10.1111/anae.15597>

| **Study conceptualisation** | |
| --- | --- |
| 1. How does this study address local research and policy priorities? | The study evaluates the National Deworming Day (NDD) initiative and community-wide mass drug administration (cMDA) in Tamil Nadu, India, specifically addressing the need for disability-inclusive health strategies to ensure no child is left behind in soil-transmitted helminth (STH) elimination efforts. |
| 1. How were local researchers involved in study design? | Local researchers from the Christian Medical College (CMC), Vellore, were integral to the conceptualization and design of the secondary analysis and the primary DeWorm3 trial, ensuring the study was culturally appropriate and aligned with local health delivery contexts. |
| **Research management** | |
| 1. How has funding been used to support the local research team(s)? | Funding from the Bill and Melinda Gates Foundation supported the local research infrastructure at CMC Vellore, enabling the implementation of trial operations and professional development for the Indian study team. |
| **Data acquisition and analysis** | |
| 1. How are research staff who conducted data collection acknowledged? | The contribution of community drug distributors (CDDs) and field workers from the local population is explicitly recognized in the manuscript for their central role in treatment delivery and community sensitization. |
| 1. How have members of the research partnership been provided with access to study data? | All research partners maintain access to the DeWorm3 trial data, which is also made available through the Vivli repository for transparency and continued collaborative use. |
| 1. How were data used to develop analytical skills within the partnership? | The partnership facilitated collaborative data analysis, where local researchers (RMR, KA, SPK) worked alongside international researchers to interpret results and apply mixed-effects logistic regression models. |
| **Data interpretation** | |
| 1. How have research partners collaborated in interpreting study data? | Interpretation of the results, particularly the nuances of geographic variation and the impact of the COVID-19 pandemic on local treatment delivery, was a joint effort between the Indian and international authors. |
| **Drafting and revising for intellectual content** | |
| 1. How were research partners supported to develop writing skills? | The manuscript was developed through a collaborative iterative process where all authors, including local researchers from various departments (Gastrointestinal Sciences, Ophthalmology, and Paediatrics), critically revised the work for intellectual content. |
| 1. How will research products be shared to address local needs? | Findings will be disseminated to local stakeholders, including community leaders and health departments in India, to inform improvements in the National Deworming Day program and disability-inclusive outreach. |
| **Authorship** | |
| 1. How is the leadership, contribution and ownership of this work by LMIC researchers recognised within the authorship? | LMIC researchers from CMC Vellore hold significant leadership roles, including the designated joint-first author (RMR) and the senior/joint last author (SSRA). |
| 1. How have early career researchers across the partnership been included within the authorship team? | Early career researchers were prioritized in key roles, such as Shanquan Chen and Rohan Michael Ramesh, who shared the lead contribution in designing and analyzing the study. |
| 1. How has gender balance been addressed within the authorship? | The authorship team demonstrates a strong gender balance, including several senior female scientists from India (KA, SSRA, BC, BK, SJ) and the UK (KH, HK) leading the research. |
| **Training** | |
| 1. How has the project contributed to training of LMIC researchers? | The DeWorm3 trial provided specialized training for local research staff on implementation science, electronic data recording. |
| **Infrastructure** | |
| 1. How has the project contributed to improvements in local infrastructure? | The DeWorm3 project strengthened the capacity of research infrastucture in Timiri and Jawadhu Hills by establishing robust community-based teams. |
| **Governance** | |
| 1. What safeguarding procedures were used to protect local study participants and researchers? | The study adhered to ethical protocols approved by the Christian Medical College (CMC) Vellore IRB, utilizing informed consent procedures tailored to local literacy levels, including oral consent and thumbprints. |
